# Improved Mouth Plates vs. Patents

**Published:** 1848-07

**Authors:** 


### IMPROVED MOUTH PLATES *VS.* PATENTS.

IN the May No. of the New Yew Dental Recordgr, we notice an article headed "Improved Mouth Plates." We sepposed it scarcely possible that, at this late day, a relic of the past should be not only revived, but actually patented; and, indeed, we can only account for the strange phenomena by recollecting that the patentee, Mr. Gilbert, is not a practicing Dentist.

We recollect, some six or eight years since, these chambers were recommended as an improvement to atmospheric plates; and, although never having adopted the practice to any extent, yet we recollect of a case where, from the intemperate habits of our patient, his gums were continually changing from the red, spungy and sowlen of the drunkard, to the hard, sound, and healthy of the sober man. They appeared to be a perfect liquor gauge, showing the amount of spirit consumed. In this case, we found air chambers or not—two sets were necessary, one for a spree and the other for the sober gentleman. We presums that Mr. Gilbert, or any other man, would find many competitors claiming this as an improvement. The natural inference with many, when these plates were first introduced, was that a chamber on either side was necessary to accomplish the object. Accurate impressions, however, with well adjusted plates, scarce ever fail to meet the views of the dentist and patient. All depends on the accuracy of this operation; yet many would be surprised on taking two impressions from the same mouth, to find that a plate which would fit one, would not fit the other. Let the dentist remember that one failure does not preclude the possibility of ultimate success. We have found it a good plan, after having drawn up a plate and it does not fit, to strike up a thick heavy plate, say of German silver, and larger than we design for the mouth; with this, then, we take another impression, using it as the cup for our wax.

We hope Mr. Gilbert will be well renumerated for his trouble and expense in perfecting his plate. We should think the comfort of a good set of teeth would well remunerate for all the trouble and expense of such a discovery, even including some $30 for letters patent.

Many of our discoveries in Dentistry, are claimed, with a good deal of pertinacity, by various members of the profession. As a general remark, the whole of it may be wound up by the following illustratinn: A. meets with an instrument which, to his taste, is a little deficient in one particular. He goes to work and remedies this defect. B. meets with the instrument thus improved—sees still another defect—remedies this and hands it over to C., under whose hands it is again improved; and thus, by the time O. gets it, it is ready for the finishing touch. This is no sooner done than O. claims all the honor, and for fear it may really be of some service to the profession and public, he gets it patented, and dazzling dreams of fortune, fame and retirement taking possession of his nightly and daily reveries. But we would ask where, in our profession, has this ever been realized? Until very recently, we supposed the dental profession as clear, if not more so, of the patent mania, as the medical profession, or any liberal science which claims for its object the good of the community. But we begin to fear that, from our gold springs to our gold foil, all will be under the *grip* of patentees.

Thank fortune dental *secrets* are getting more and more scarce every year; and we begin to hope we may live to see dental knavery buried, and the last secret, credulous folly and hoarded ignorance gloats open to fill her pocket, completely exposed to public view.—CIN. ED.

